# Genetic Diversity and Population Genetic Structure of *Setosphaeria turcica* From Sorghum in Three Provinces of China Using Single Nucleotide Polymorphism Markers

**DOI:** 10.3389/fmicb.2022.853202

**Published:** 2022-03-02

**Authors:** Linkai Cui, Junli Deng, Linxi Zhao, Yanhong Hu, Tingli Liu

**Affiliations:** ^1^College of Horticulture and Plant Protection, Henan University of Science and Technology, Luoyang, China; ^2^Jiangsu Key Laboratory for Horticultural Crop Genetic Improvement, Institute of Leisure Agriculture, Jiangsu Academy of Agricultural Sciences, Nanjing, China

**Keywords:** *Setosphaeria turcica*, genetic diversity, population genetic structure, mating type, SNP molecular marker

## Abstract

*Setosphaeria turcica* is a heterothallic fungus that is the causal agent of northern leaf blight (NLB), which is a devastating foliar disease of sorghum and maize. Despite of its adversary to crop production, little is known about the genetic diversity and population genetic structure of this pathogen from sorghum. In this study, we explored the utilization of single nucleotide polymorphism (SNP) molecular markers and three mating type-specific primers to analyze the genetic diversity, population genetic structure, and mating type distribution of 87 *S. turcica* isolates that had been collected in sorghum production areas from three provinces, including Henan, Shaanxi, and Shanxi in China. The populations are featured with moderate genetic diversity and relatively equal mating type distribution of MAT1-1 and MAT1-2. The genetic differentiation was significant (*p* < 0.05) among different populations except those from Henan and Shanxi provinces that showed particularly frequent gene flow between them. Neither the maxinum likelihood phylogenetic tree, nor principal coordinate analysis, nor genetic structure analysis was able to completely separate the three populations. The relatively low genetic distance and high genetic identification were also observed among the three populations. Nevertheless, the genetic variation within populations was the major source of variation as revealed by AMOVA analysis. The findings of this study have improved our current understanding about the genetic diversity, population genetic structure, and the distribution of mating type of *S. turcica*, which are useful for unraveling the epidemiology of NLB and developing effective disease management strategies.

## Introduction

Sorghum (*Sorghum bicolor*) is an important crop with multiple uses for food, brewing, fodder, and biofuel applications in many countries in the world (Lu and Dahlberg, 2001); and it is reemerging to prominence as an alternative crop for a number of high yielding crops, such as corn, due to its stronger drought tolerance, especially in the current circumstances with increasing occurrence of drought and change of weather patterns (Nieuwoudt et al., 2018). However, the production of sorghum is severely constrained by the infestation of northern leaf blight (NLB) which is caused by a pathogen known as *Setosphaeria turcica* (Luttr.) Leonard and Suggs (1974); anamorph: *Exserohilum turcicum* (Pass.) Leonard and Suggs (1974). Apart from sorghum, NLB is also a devastating foliar disease of maize and some other related grass species wherever they are cultivated (Leonard and Suggs, 1974; Tang et al., 2015). NLB is very prevalent, spreading more widely, especially in the environment, such as warm and humid conditions, that is conductive to the development of the disease (Ferguson and Carson, 2004), which causes considerable economic destruction. The disease mainly infects sorghum leaves, whose typical symptoms are long fusiform, light brown to brown in the center, purple at the edge, and irregular ring patterns can be seen in the early stage (Dai et al., 2021). Upon NLB infection, the yield of sorghum could be severely compromised as the result of reduction in photosynthetic potential of the plant.

As a heterothallic ascomycete, the individual isolate of *S. turcica* cannot mate with itself, and its sexual reproduction requires two isolates with complementary mating type genes. The perfect stage (teleomorph or sexual stage) of *S. turcica* was first described in 1958 (Luttrell, 1958), which is controlled by a specific mating type (MAT) gene locus that may appear in two alternative allele forms, namely, MAT1-1 and MAT1-2 (Luttrell, 1958). Although rarely occurs in the field, the sexual stage of *S. turcica* is can be observed under laboratory conditions (Luttrell, 1958; Fan et al., 2007). Previous research results have revealed that the pathogenicity and parasitic adaptability of some sexual progenies are significantly enhanced after sexual reproduction (Guo et al., 2013). In addition, various races of *S. turcica* with different mating type could mate randomly to produce new races of different pathogenicity (Hou et al., 2006; Fan et al., 2007). It is generally recognized that the genetic variability and pathogenicity of *S. turcica* are the key factors that affect host-plant resistance and develop feasible strategies for disease management (Sree et al., 2012). This necessitates studies in these areas to better understand the relationship between genetic diversity and mating type of *S. turcica*.

Molecular markers have been successfully used to analyze genetic diversity, population genetic structure, and mating type distribution of *S. turcica* isolates, such as universally primed polymerase chain reaction (UP-PCR), random amplified polymorphic DNA (RAPD), simple sequence repeat (SSR), and sequence-related amplified polymorphism (SRAP). RAPD molecular markers were developed to analyze genetic diversity and population genetic structure of 264 *S. turcica* isolates from four different continents, which displayed the variability in the genetic diversity and mating type distribution of different regions and states (Borchardt et al., 1998). This was corroborated by further studies by using UP-PCR molecular markers that the pathogenic specialization of *S. turcica* isolates was more correlated with genetic diversity than geographic distribution (Tang et al., 2015). Abundant genetic diversity among various *S. turcica* isolates from different geographic locations was also demonstrated by SSR molecular marker (Li et al., 2019) and SRAP molecular markers (Ma et al., 2020a). A significant correlation between mating types and cluster groups of *S. turcica* isolates was also established by using SSR markers (Li et al., 2019).

Despite the genetic diversity of *S. turcica* is well documented, a systematic and comprehensive analysis of the genetic structure and genetic diversity of the pathogen by using the single nucleotide polymorphism (SNP) molecular marker is lacking. SNP is the third-generation molecular marker which is characteristic of high stability, large number, wide distribution, and the ability to detect the most frequent type of DNA variation (Tang et al., 2012). What’s more, there are relatively fewer studies on *S. turcica* from sorghum compared to *S. turcica* from corn in China, because sorghum is not normally considered as a food crop there. In the present study, we analyzed the genetic diversity and population genetic structure of 87 *S. turcica* isolates from Henan, Shaanxi, and Shanxi provinces in China, by using SNP molecular markers for the first time. We aim to improve our current understanding the genetic variation and evolution of *S. turcica*, which may provide useful information for the disease management of NLB in sorghum.

## Materials and Methods

### Collection and Isolation of Samples

**Figure 1:**
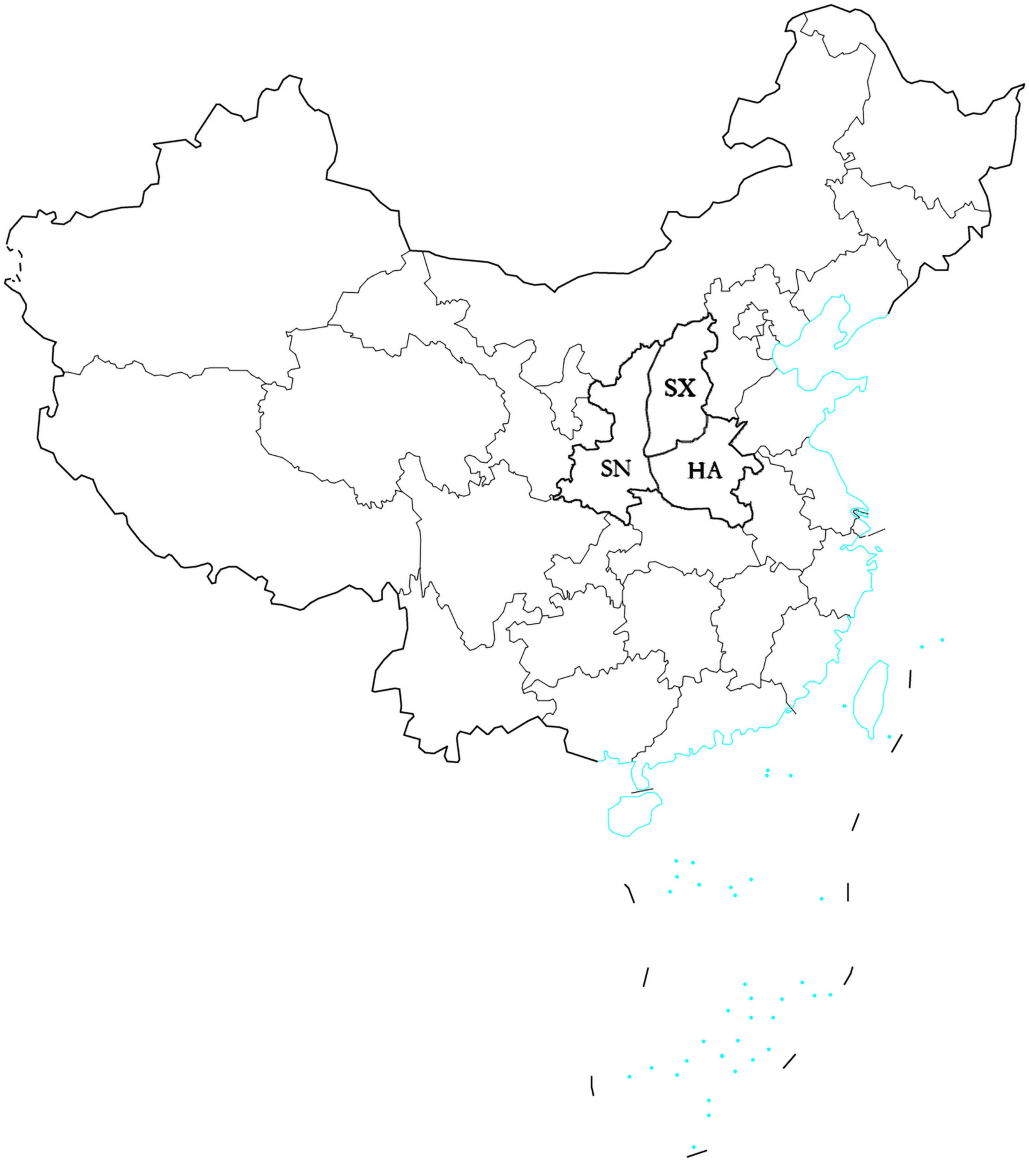
Map showing the location of three provinces in which *Setosphaeria turcica* isolates were collected. The blue regions denote coastlines or islands. HA, Henan province; SX, Shanxi province; SN, Shaanxi province.

In 2019, the diseased leave with typical symptom were collected randomly at the middle and late stages of sorghum growth from Henan, Shanxi, and Shaanxi provinces of China (Figure 1). A total of 87 isolates were obtained. Thirty isolates were from Henan province, thirty isolates were from Shanxi province, and 27 isolates were from Shaanxi province (Table 1).

**Table 1:** Information of 87 *Setosphaeria turcica* isolates from 3 provinces in China, obtained from sorghum samples collected in 2019.

Isolates	Locations	Isolates	Locations
HAS19-02	Luoyang, Henan	SNS19-16	Weinan, Shaanxi
HAS19-03	Luoyang, Henan	SNS19-18	Weinan, Shaanxi
HAS19-04	Luoyang, Henan	SNS19-19	Weinan, Shaanxi
HAS19-05	Luoyang, Henan	SNS19-20	Weinan, Shaanxi
HAS19-06	Luoyang, Henan	SNS19-21	Weinan, Shaanxi
HAS19-07	Luoyang, Henan	SNS19-22	Weinan, Shaanxi
HAS19-09	Sanmenxia, Henan	SNS19-23	Weinan, Shaanxi
HAS19-10	Sanmenxia, Henan	SNS19-25	Weinan, Shaanxi
HAS19-11	Sanmenxia, Henan	SNS19-26	Weinan, Shaanxi
HAS19-13	Sanmenxia, Henan	SNS19-27	Weinan, Shaanxi
HAS19-14	Sanmenxia, Henan	SNS19-28	Weinan, Shaanxi
HAS19-15	Sanmenxia, Henan	SNS19-30	Weinan, Shaanxi
HAS19-16	Sanmenxia, Henan	SNS19-31	Weinan, Shaanxi
HAS19-18	Sanmenxia, Henan	SXS19-01	Yuncheng, Shanxi
HAS19-19	Sanmenxia, Henan	SXS19-02	Yuncheng, Shanxi
HAS19-21	Sanmenxia, Henan	SXS19-03	Yuncheng, Shanxi
HAS19-22	Sanmenxia, Henan	SXS19-04	Yuncheng, Shanxi
HAS19-24	Sanmenxia, Henan	SXS19-05	Yuncheng, Shanxi
HAS19-25	Sanmenxia, Henan	SXS19-06	Yuncheng, Shanxi
HAS19-27	Sanmenxia, Henan	SXS19-08	Yuncheng, Shanxi
HAS19-29	Luoyang, Henan	SXS19-09	Yuncheng, Shanxi
HAS19-31	Luoyang, Henan	SXS19-10	Yuncheng, Shanxi
HAS19-32	Luoyang, Henan	SXS19-11	Yuncheng, Shanxi
HAS19-33	Luoyang, Henan	SXS19-13	Yuncheng, Shanxi
HAS19-35	Luoyang, Henan	SXS19-14	Yuncheng, Shanxi
HAS19-36	Luoyang, Henan	SXS19-15	Yuncheng, Shanxi
HAS19-37	Luoyang, Henan	SXS19-18	Yuncheng, Shanxi
HAS19-38	Luoyang, Henan	SXS19-19	Yuncheng, Shanxi
HAS19-39	Luoyang, Henan	SXS19-20	Yuncheng, Shanxi
HAS19-40	Luoyang, Henan	SXS19-22	Yuncheng, Shanxi
SNS19-01	Xi’an, Shaanxi	SXS19-23	Yuncheng, Shanxi
SNS19-02	Xi’an, Shaanxi	SXS19-24	Yuncheng, Shanxi
SNS19-03	Huayin, Shaanxi	SXS19-27	Yuncheng, Shanxi
SNS19-04	Huayin, Shaanxi	SXS19-28	Yuncheng, Shanxi
SNS19-05	Huayin, Shaanxi	SXS19-29	Yuncheng, Shanxi
SNS19-07	Huayin, Shaanxi	SXS19-30	Yuncheng, Shanxi
SNS19-08	Xianyang, Shaanxi	SXS19-31	Yuncheng, Shanxi
SNS19-09	Xianyang, Shaanxi	SXS19-32	Linfen, Shanxi
SNS19-10	Xianyang, Shaanxi	SXS19-33	Linfen, Shanxi
SNS19-11	Xianyang, Shaanxi	SXS19-34	Linfen, Shanxi
SNS19-12	Xianyang, Shaanxi	SXS19-35	Linfen, Shanxi
SNS19-13	Xianyang, Shaanxi	SXS19-36	Yuncheng, Shanxi
SNS19-14	Xianyang, Shaanxi	SXS19-37	Yuncheng, Shanxi
SNS19-15	Xianyang, Shaanxi		

*S. turcica* were isolated by using the tissue separation methods. In brief, the infected sorghum leaves were cut into 2–3 mm pieces, which were disinfected with 75% alcohol for 20 s (sec), followed by 3% sodium hypochlorite for 2–3 min (min), and washed with sterile water for three times, prior to being transferred onto the potato dextrose agar (PDA) medium containing rifampicin (RFP) at 50 μg/ml and incubated at 25°C for 3–5 days (d). All the cultures were checked for conidiophore and conidium under microscopy to verify that isolates were *S. turcica* before being stored at 4°C until further use.

### DNA Extraction

*Setosphaeria turcica* isolates were cultured in liquid potato dextrose broth for 3 d at 25°C to collect mycelium, from which total DNA was extracted using a rapid blood genomic DNA isolation kit (Sangon, Shanghai, China), following the manufacturer’s instruction. The quality and concentration of the obtained DNA was determined by using Qubit 2.0 fluorometer (Thermo Fisher Scientific, Waltham, MA, United States).

### Primer Design

A total of 172,067 potential SNP loci were detected through the whole-genome resequencing of 15 *S. turcica* isolates^1^ using the GATK software, from which 80 polymorphic SNP loci were selected based on a minimum distance of 300 kb between SNP loci. The primers were designed by the online tool Batchprimer 3^2^ to amplify the target sequence in the range of 140 bp to 270 bp, the primer length was between 18 bp and 35 bp, and G/C content in any primer was between 35 to 65%. Finally, 77 SNP primer pairs were successfully designed (Supplementary Table S1), which were commercially synthesized by Sangon Biotech (Shanghai, China).

### Multiplex PCR and Sequencing

A panel consisting of 77 target SNP sites was designed, and libraries were prepared by using two-step PCR. The PCR reaction consisted of 1 μl each of the forward and reverse amplicon PCR primers (10 μm), 15 μl kapa hifi 2 × PCR ready mix (Kapa Biosystems, Cape Town, South Africa), 2 μl genomic DNA (10 ng/μl), and sterile water to 25 μl. PCR reaction was executed in a T100^™^ Thermal Cycler (BIO-RAD, Hercules, CA, United States) with the following conditions: an initial denaturation at 98°C for 5 min, first 8 cycles of denaturation at 98°C for 30 s, annealing at 50°C for 30 s, elongation at 72°C for 30 s, and then 25 cycles of denaturing at 98°C for 30 s, annealing at 66°C for 30 s, elongation at 72°C for 30 s and a final extension at 72°C for 5 min, eventually held at 4°C. The PCR products were fractionated by using gel electrophoresis on 1% (w/v) agarose gels in TBE buffer (Tris, boric acid, EDTA), stained with ethidium bromide (EB) prior to visualization under UV light in order to verify the size of the desired PCR product. The PCR product was then isolated from the gel and recovered using AMPure XP magnetic beads (Beckman Coulter, Brea, CA, United States). The second round of PCR reaction was performed using the first round of PCR product as a template to obtain a sequencing library with molecular tags. The second round of PCR reaction consisted of DNA (10 ng/μl) 2 μl, universal P7 primer (10 μm) 1 μl, universal P5 primer (10 μm) 1 μl, 2× PCR Ready Mix 15 μl. The condition of PCR reaction: an initial denaturation at 98°C for 3 min, then 5 cycles of denaturing at 94°C for 30 s, annealing at 55°C for 30 s, elongation at 72°C for 30 s and a final extension at 72°C for 5 min. When the second round of PCR reaction was completed, the PCR product was also purified and recovered using AMPure XP magnetic beads. Following the preparation of the DNA sequencing libraries, paired-end sequencing of the library was performed on the HiSeq XTen sequencers (Illumina, San Diego, CA, United States).

Raw reads were filtered and having adaptor sequence removed by Cutadapt (v 1.2.1), and the low-quality bases from reads 3′ to 5′ (*Q* < 20) removed by PRINSEQ-lite (v 0.20.3). The remaining clean data were then mapped to the reference genome (GenBank assembly accession: GCA_013390295.1) by BWA (version 0.7.13-r1126) with default parameters. Samtools (version: 0.1.18) was used to calculate each genotype of the target locus. When the read frequency of a major allele was higher than 80%, it was described as homozygous, and those loci with missing data were discarded.

### Data Analysis

The SNP sites with high level of polymorphisms, but without missing data, were selected for further analysis. Arlequin 3.11 (Ma et al., 2020b) was used to analyze gene flow and the genetic variation of the three population, the genetic differentiation coefficient (Fst), and the number of migrants per generation (Nm) satisfied the following equation: Nm = (1-Fst)/4 Fst (Jimenez et al., 1999). Analysis of molecular variance (AMOVA) was also calculated through the Arlequin 3.11 software (Ma et al., 2020b). A range of parameters, including the observed number of alleles (Na), the number of effective alleles (Ne), Nei (1973) gene diversity (h), Shannon’s information index (I), Nei’s genetic identity (Nei’s I) and genetic distance (Nei’s D) were all calculated by POPGENE (Yeh et al., 1997). A phylogenetic tree was constructed by using maximum likelihood method with 1,000 bootstrap in Mega7 (Nei and Kumar, 2000; Kumar et al., 2016). Genetic distance (GD) and Principal Coordinate Analysis (PCoA) were analyzed by using GenALEx v6.5 (Peakall and Smouse, 2012). Population genetic structure was assessed by Structure 2.3.4 (Pritchard et al., 2000). Every possible cluster (*K*) ranges from 1 to 10 with 10 runs at 10,000 burn-in followed by 100,000 Markov Chain Monte Carlo simulations. The optimal value of *K* was acquired by online tool Structure Harvester^3^ after obtaining the result from Structure (Earl, 2012). Eventually, population genetic structure was visualized by CLUMPP and DISTRUCT (Rosenberg, 2004; Jakobsson and Rosenberg, 2007).

### Determination of Mating Type

The mating type of 87 isolates was determined by using mating type-specific primers which were designed by Haasbroek et al. (2014). The same reverse primer, MAT_CommonR (5′-AATGCGGACACGGAATAC -3′), was used in combination with each of the two different forward primers: MAT_1-1F (5′-CTCGTCCTTGGAGAAGAATATC-3′) and MAT_1-2F (5′-GCTCCTGGACCAAATAATACA-3′). A PCR fragment in the length of 608 bp was amplified from the MAT1-1 locus and the other fragment in the length of 393 bp was from the MAT1-2 locus. The PCR was performed using a mix containing 12.5 μl 2 × PCR Taq Master Mix (Biotechnologies Co., Ltd., Beijing, China), 1 μm mixture of three primers, 100 ng DNA, with sterile water adding up to 25 μl. The program was carried out first with initial denaturation at 94°C for 2 min, and then 30 cycles of denaturation at 94°C for 30 s, annealing at 61°C for 30 s, elongation at 72°C for 1 min; ending with a final extension at 72°C for 10 min, and finally held at 4°C. The PCR products were analyzed on a 1% (w/v) agarose gel electrophoresis running for 30 min at 100 V, which were then stained with ethidium bromide (EB) and visualized under UV light. The ratios between MAT1-1 and MAT1-2 from three different geographical population were subjected to a 2 test using IBM SPSS Statistics 20 (International Business Machines Corporation, Armonk, NY, United States; Zeng et al., 2021) to estimate divergence from the expected ratio of 1:1 at the *p* < 0.05 level.

## Results

### Genetic Diversity Analysis

Among the 77 SNP loci analyzed, 41 loci containing missing data or monomorphisms were discarded, while the remaining 36 SNP loci with high-quality and informative polymorphisms were retained to assess the genetic diversity of 87 *S. turcica* isolates (Table 2).

**Table 2:** Characteristics of 36 SNP markers used for genetic diversity of *Setosphaeria turcica* isolates in China.

Locus	Ref	Alt	Allele I^1^	Allele II^2^	Na^3^	Ne^4^	h^5^	I^6^
L01	C	T	0.7356	0.2644	2	1.6365	0.3890	0.5776
L02	A	C	0.6897	0.3103	2	1.7484	0.4281	0.6194
L03	C	T	0.4828	0.5172	2	1.9976	0.4994	0.6926
L04	A	G	0.6092	0.3908	2	1.9090	0.4762	0.6691
L05	G	C	0.4023	0.5977	2	1.9264	0.4809	0.6739
L07	G	A	0.3563	0.6437	2	1.8474	0.4587	0.6513
L08	G	A	0.4138	0.5862	2	1.9423	0.4851	0.6782
L09	C	T	0.5172	0.4828	2	1.9976	0.4994	0.6926
L11	C	G	0.6552	0.3448	2	1.8243	0.4518	0.6442
L13	G	A	0.6437	0.3563	2	1.8474	0.4587	0.6513
L14	G	A	0.1379	0.8621	2	1.3120	0.2378	0.4012
L15	T	C	0.6552	0.3448	2	1.8243	0.4518	0.6442
L18	G	A	0.7241	0.2759	2	1.6653	0.3995	0.5890
L19	G	T	0.1494	0.8506	2	1.3408	0.2542	0.4217
L22	G	A	0.5862	0.4138	2	1.9423	0.4851	0.6782
L26	C	A	0.8391	0.1609	2	1.3700	0.2700	0.4412
L29	G	C	0.6552	0.3448	2	1.8243	0.4518	0.6442
L30	C	T	0.6092	0.3908	2	1.909	0.4762	0.6691
L33	A	G	0.7471	0.2529	2	1.6073	0.3779	0.5655
L36	T	C	0.5977	0.4023	2	1.9264	0.4809	0.6739
L37	C	T	0.9195	0.0805	2	1.1737	0.1480	0.2799
L39	C	G	0.8621	0.1379	2	1.3120	0.2378	0.4012
L40	G	A	0.4023	0.5977	2	1.9264	0.4809	0.6739
L46	G	A	0.8391	0.1609	2	1.3700	0.2700	0.4412
L48	C	T	0.5977	0.4023	2	1.9264	0.4809	0.6739
L50	T	C	0.0115	0.9885	2	1.0233	0.0227	0.0628
L55	C	A	0.7356	0.2644	2	1.6365	0.3890	0.5776
L58	G	A	0.4138	0.5862	2	1.9423	0.4851	0.6782
L59	G	C	0.7586	0.2414	2	1.5779	0.3662	0.5527
L60	G	A	0.5057	0.4943	2	1.9997	0.4999	0.6931
L61	A	G	0.3333	0.6667	2	1.8000	0.4444	0.6365
L62	G	A	0.4943	0.5057	2	1.9997	0.4999	0.6931
L63	C	T	0.7126	0.2874	2	1.6937	0.4096	0.5998
L64	T	A	0.6437	0.3563	2	1.8474	0.4587	0.6513
L66	G	C	0.2529	0.7471	2	1.6073	0.3779	0.5655
L70	C	T	0.4023	0.5977	2	1.9264	0.4809	0.6739
Mean					2	1.7267	0.4046	0.5870

1 *Allele I = Ref.*

2 *Allele II = Alt.*

3 *Na = Observed number of alleles.*

4 *Ne = Effective number of alleles.*

5 *h = Nei’s gene diversity.*

6 *I = Shannon’s information index.*

The genetic diversity information of the 36 SNP loci was provided in Table 2. The observed number of alleles (Na) at each SNP locus was 2. The average number of effective alleles (Ne) was 1.7267, ranging from 1.0233 to 1.9997. The minor allele frequency (MAF) ranged from 0.0115 to 0.4943, with about 80% of the markers having MAF > 0.2. Nei’s gene diversity (h) ranged from 0.0227 to 0.4999, with the mean of 0.4046. Shannon’s information index (I) varied from 0.0628 to 0.6931, with the mean of 0.587. In particular, the SNP locus L50 showed the lowest value, whereas the polymorphisms at L60 and L62 were the most abundant.

The genetic diversity among the three populations was summarized in Table 3. The values of the observed number of alleles (Na) of the three populations ranged from 1.9444 to 1.9722 with the mean of 1.9537. The average number of effective alleles (Ne) was 1.6413, varying from 1.5774 to 1.7541. Nei’s gene diversity (h) ranged from 0.3430 to 0.4053 with the mean of 0.3668. Shannon’s information index (I) ranged from 0.5140 to 0.5793, with the mean of 0.5387. Although the variations among the three populations were not particularly large, relatively speaking, the genetic diversity of the Henan population was the lowest, and that of the Shaanxi population was the highest.

**Table 3:** Estimates of genetic diversity of three populations of *Setosphaeria turcica* based on 36 SNP markers.

Pop	Na^1^	Ne^2^	h^3^	I^4^
Henan	1.9722	1.5774	0.3430	0.5140
Shaanxi	1.9444	1.7541	0.4053	0.5793
Shanxi	1.9444	1.5924	0.3520	0.5229
Mean	1.9537	1.6413	0.3668	0.5387

1 *Na = Observed number of alleles.*

2 *Ne = Effective number of alleles.*

3 *h = Nei’s (1973) gene diversity.*

4 *I = Shannon’s information index.*

### Population Genetic Structure Analysis

**Figure 2:**
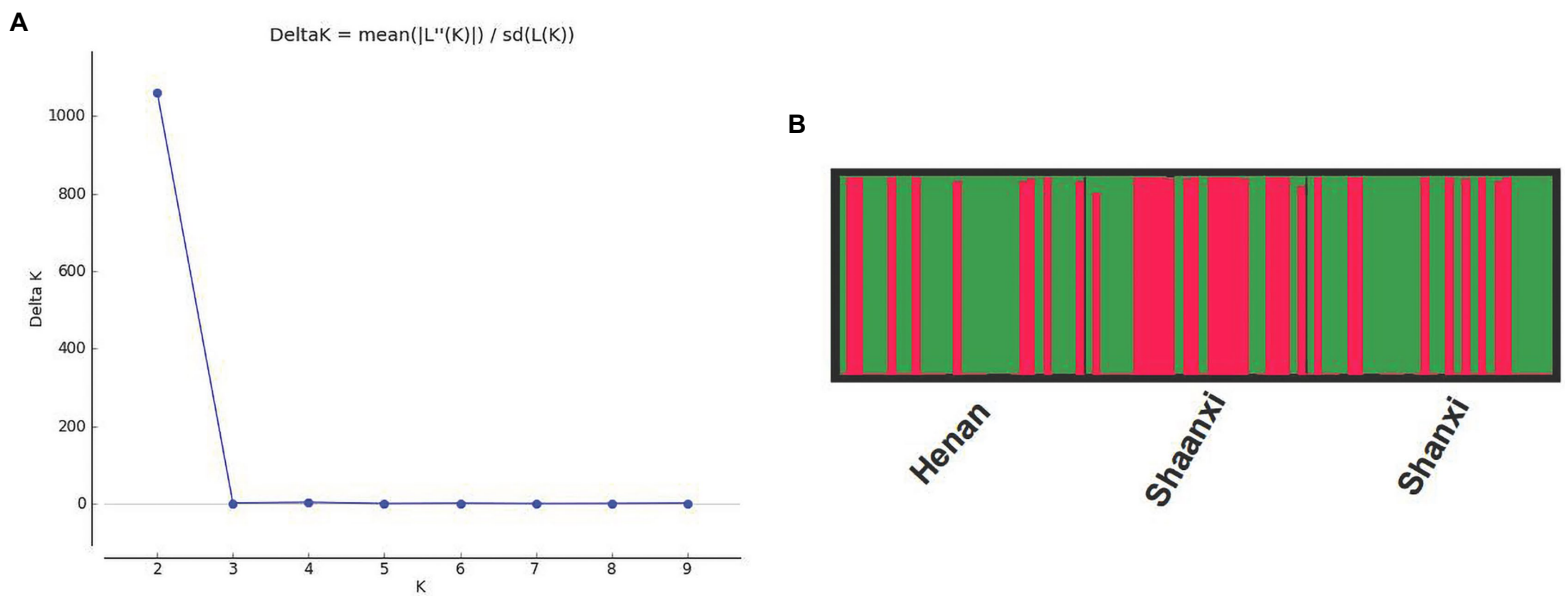
Results of the STRUCTURE analysis of the 87 isolates of *Setosphaeria turcica* from China using 36 SNP markers. **(A)** DeltaK curve showing evidence for two genetic clusters. **(B)** Different colors represent different genetic clusters (*K* = 2) with each vertical line representing an isolate. Thick black lines separate different geographic populations. HA, SX, and SN represent the Henan, Shanxi, and Shaanxi geographic populations, respectively.

The optimum *K* value was obtained by analyzing the three populations using the online tool Structure Harvester. When the Δ*K* value was the biggest, there was the optimal number of genetic clusters. We could observe that the best *K* value was 2, revealing that all isolates came from two genetic clusters (Figure 2A). In these three populations, each population consisted of two genetic clusters, termed as Cluster I and Cluster II, but the main source of clusters in every population was different (Figure 2B). The Henan and Shanxi populations were dominated by Cluster I, while the main constituent of Shaanxi population was Cluster II. Most of the isolates were classified into one of the two clusters, whereas the remaining small proportions of the isolates formed a mixture of the two clusters. It appears clear that although there was no particularly significant correlation between genetic clusters and geographic subgroup, genetic divergence among the Henan, Shanxi, and Shaanxi populations does exist.

### Phylogenetic Analysis

**Figure 3:**
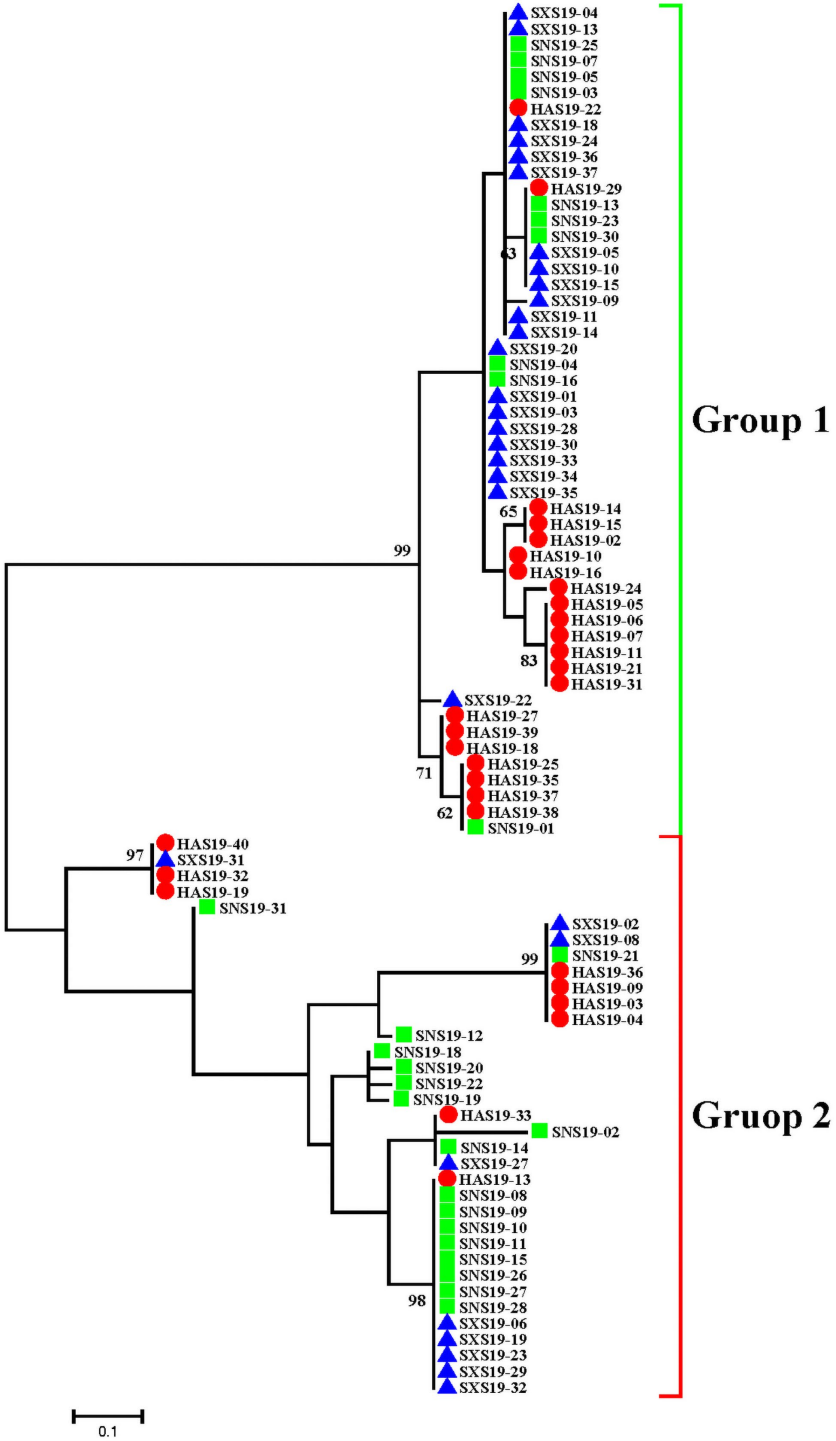
Phylogenetic tree of *Setosphaeria turcica* based on maximum likelihood method for 87 isolates from three geographic populations in China. The red circle, green square, and blue triangle represented the Henan, Shaanxi, and Shanxi populations, respectively.

The relationship of the three populations was also demonstrated by phylogenetic tree of maximum likelihood means, which corroborated the population genetic STRUCTURE analysis (Figure 3). Based on 0.08 genetic distance, 87 isolates were divided into two groups. There were 52 isolates in group 1, including 21 isolates from Henan, 21 isolates from Shanxi, and 10 isolates from Shaanxi. There were 35 isolates in group 2, including 9 isolates from Henan, 9 isolates from Shanxi, and 17 isolates from Shaanxi. Though each group contained three subpopulations, the proportions of the three subpopulations in each group were variable. In Group 1, Henan and Shanxi populations accounted for a larger proportion, and were the main subpopulations, whereas Shaanxi population was the main subpopulation in Group 2. Therefore, although there was no particularly significant correlation between genetic groups and geographic origin, there might be a certain genetic distance between Henan, Shanxi, and Shaanxi populations, together with genetic differentiation to some extent.

### Principal Coordinate Analysis

**Figure 4:**
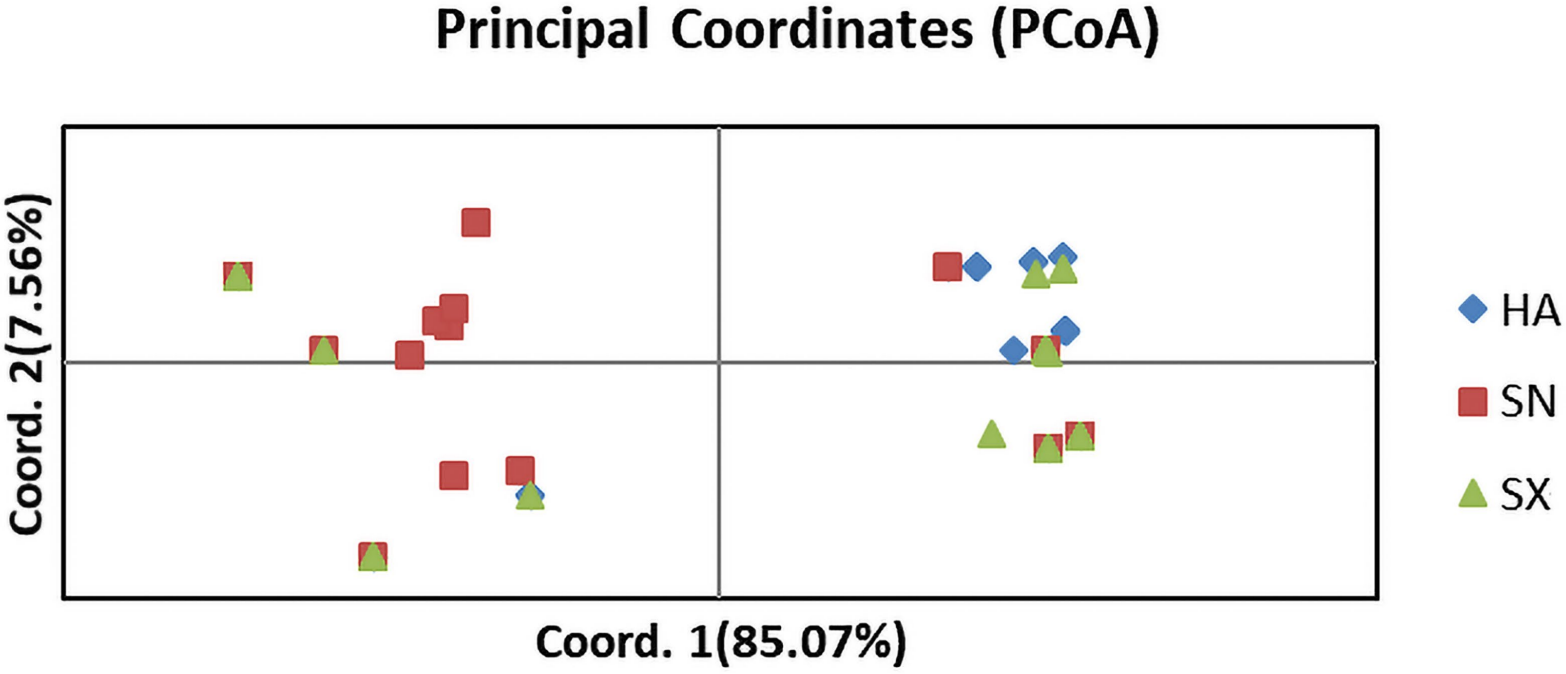
Principal component analysis (PCoA) based on SNP marker data for 87 isolates collected from three geographic populations in China. Individuals within the same population are marked using the same color and symbol. HA, SN and SX represent isolates from the geographic populations of Henan, Shaanxi and Shanxi, respectively. The abscissa component accounted for 85.07% and the ordinate component accounted for 7.56%.

The analysis methods of maxinum likelihood (ML) phylogenetic tree, population STRUCTURE, and principal coordinate analysis (PCoA) are complementary in this study. PCoA based on the paired genetic distance matrix also elucidated the population genetic structure. As shown in Figure 4, the PCoA analysis primarily contained two components, among which the abscissa component accounted for 85.07% and the ordinate component accounted for 7.56%. The sum of these two components exceeded 90%, which could better explain the population genetic structure. It appears that there are no distinct separations, but rather they are often mixed together to various extent. Such a result is a manifestation that gene exchanges among the three populations are relatively frequent and the genetic diversity is rather limited.

### Analysis of Genetic Differentiation Between Populations

The genetic differentiation coefficient (Fst) was calculated to compare the three populations. Among Henan/Shaanxi, Henan/Shanxi, and Shaanxi/Shanxi, the Fst values were 0.1581, 0.0385, and 0.1208, respectively; the number of migrants per generation (Nm) values was 1.3253, 6.4885, and 1.8356, respectively (Table 4). The Fst values ranging from 0 to 0.05, 0.05 to 0.15, 0.15 to 0.25, and more than 0.05 represent low, moderate, high, and great genetic differentiations, respectively (Hartl and Clark, 1997). The value of Nm greater than 4 indicated that gene exchanges between the two populations are frequent (Jimenez et al., 1999). Because the gene exchanges between the Henan/Shaanxi populations were the most frequent, resulting in the lowest degree of genetic differentiation between these two populations. The genetic differentiation coefficient clearly displayed that genetic divergence was significant among populations Henan/Shaanxi and Shaanxi/Shanxi. Such a result indicated that there was a certain degree of genetic differentiation among the three populations, and gene exchanges were relatively frequent.

**Table 4:** Pairwise matrices of genetic differentiation coefficient (Fst, below the diagonal) and the number of migrants per generation (Nm, above the diagonal) among *Setosphaeria turcica* populations in China.

Population	Henan	Shaanxi	Shanxi
Henan		1.3253	6.4885
Shaanxi	0.1581		1.8356
Shanxi	0.0385	0.1208	

### Genetic Distance and Genetic Identity Between Populations

Nei’s genetic distance (Nei’s D) and genetic identity (Nei’s I) mainly analyzed the genetic relationship between populations, as tabulated in Table 5. Nei’s I varied from 0 to 1, Nei’s D ranged from 0 to infinity. The closer the Nei’s I value was to 1, the higher the genetic similarity between the two populations will be; and the larger the Nei’s D value, the greater the genetic distance between the two populations will be (Liu and Mundt, 2020). Among the three groups, Henan/Shanxi had the lowest Nei’s D values, and Henan/Shaanxi had the highest values. On the other hand, Nei’I value of Henan/Shanxi was the highest, being the closest to 1, while Henan/Shaanxi had the lowest Nei’I value. Therefore, the genetic similarity between the Henan and Shanxi populations was the highest, and correspondingly the genetic distance was the closest and the communication between them was the most frequent, while the genetic similarity between Henan and Shaanxi, between Shaanxi and Shanxi were relatively lower.

**Table 5:** Pairwise matrices of Nei’s genetic identify (above the diagonal) and Nei’s genetic distance (below the diagonal) among *Setosphaeria turcica* population in China.

Population	Henan	Shaanxi	Shanxi
Henan		0.8635	0.9596
Shaanxi	0.1468		0.8922
Shanxi	0.0413	0.1140	

### Analysis of Molecular Variation

Analysis of molecular variance (AMOVA) revealed that the variation within and between the population accounted for approximately 89.23 and 10.78% of the total variation, respectively (Table 6). Such a result clearly demonstrated that the genetic variation within the populations, rather than between populations, was the major source of total variation.

**Table 6:** Analysis of molecular variance (AMOVA) for *Setosphaeria turcica* populations in China.

Source of variation	Degree of freedom	Sum of squares	Variance components	Percent of variation (%)
Among pops	2	61.30	0.82	10.78
Within pops	84	572.26	6.81	89.23
Total	86	633.56	7.64	100.00

### Identification of Mating Type and Ratios

The mating types of the 87 *S. turcica* isolates were investigated by using multiplex PCR analysis with mating type-specific primers (Table 7). As a result, 39 isolates were determined as MAT1-1, and the remaining 48 isolates were determined as MAT1-2, accounting for 44.83 and 55.17% of the total isolates, respectively. The frequencies of mating type did not deviate significantly from a 1:1 ratio by the *χ*^2^ test (0.1 ≤ *p* ≤ 1.0). In the Henan population, 15 isolates were MAT1-1 and 15 were MAT1-2, representing a perfect ratio of 1:1. However, this was not the case in the Shaanxi and Shanxi populations, especially in Shanxi population, about 73% of isolates were identified as MAT1-2. Moreover, the isolates of *S. turcica* containing both MAT1-1 and MAT1-2 mating types were not found.

**Table 7:** Analysis of molecular variance (AMOVA) for *Setosphaeria turcica* populations in China.

Pop	MAT1-1	MAT1-2	*χ* ^2^	*P*
Henan	15	15	0.000	1.0
Shaanxi	16	11	0.475	0.5
Shanxi	8	22	3.455	0.1
Total	39	48	0.469	0.5

## Discussion

In this study, the population genetic diversity, genetic structure, and distribution of mating type of *S. turcica* isolates sampled from Henan, Shaanxi and Shanxi provinces in China were investigated by using SNP markers and mating type-specific primers. Population genetic diversity is prerequisite for evaluating the status of biological resources. Nei’s genetic diversity (h) and Shannon’s information index (I) are important parameters for measuring biological genetic diversity (Lu et al., 2009). Moderate level of genetic diversity in these three population was found as indicated by Shannon’s information index (0.51–0.57), which is somewhat incongruent with previous studies on the genetic diversity of *S. turcica* isolates. Ma et al. (2020a) and Dai et al. (2021), respectively, attained low Shannon’s information index of 0.20 to 0.37 and 0.07 to 0.33, indicating low genetic diversity among different populations. Whereas Human et al. (2016) and Nieuwoudt et al. (2018) both achieved high level of genetic diversity with Shannon’s information index of 0.87 to 0.90 and 0.66 to 0.94. It is conceivable that genetic diversity may vary among different geographic populations.

The population genetic STRUCTURE and ML phylogenetic tree both separated the three populations into two groups, and the variations among the Henan, Shanxi, and Shaanxi populations is a manifestation that there was no particularly significant connection between genetic groups and the geographic origin of *S. turcica* isolates. Such a finding is consistent with a previous study that SSR groups were not significantly correlated with physiological races or geographic locations (Li et al., 2019). The same was true for studies on the relationship between genetic diversity and geographic regions in *S. turcica* in South Africa (Human et al., 2016), and elsewhere (Gao et al., 2011). However, contrary findings have also been reported (Borchardt et al., 1998). We speculate that the airborne transmission of conidia through wind or other media and the proximity of the three provinces might well be attributable for the observation made in this study that the genetic group is not significantly related to its geographic origin among the *S. turcica* isolates. In addition, the estimation of Nei’s genetic identity and genetic distance are reliable parameters for assessing the genetic relationship between populations. The relatively high level of genetic identity (0.9596) between Henan and Shanxi population also illustrated the similarity of the two subgroups, further corroborating the above findings.

Gene flow (Nm) can reveal genetic infiltration between populations, reduce geographic differentiation and cause the homogenization of the genetic characteristics between populations (Rousset, 1997); Fst can be used to clarify the level of genetic variation between populations and is also a vital parameter for understanding the evolutionary history of the population. Hedrick (2005) reported that the larger the value of Fst is, the greater is the level of genetic divergence between populations. In this study, we identified a relatively high level of gene flow (6.2385) of *S. turcica* isolates between Henan and Shanxi, indicating frequent gene exchange between these two populations. The value of Fst between Henan/Shaanxi (0.1573) and Shanxi/Shaanxi (0.1202) was greater than Henan/Shanxi (0.0385), demonstrating relatively moderate genetic divergence between these populations, which was lower than that previously reported by Dai et al. (2021). In addition, the result of the AMOVA analysis demonstrated the genetic variation within populations, rather than those between populations, was the major source of genetic variation, which was consistent with previous reports by Li et al. (2019) and Ma et al. (2020a).

By using type-specific PCR primers, we were able to identify two mating types, including MAT1-1 and MAT1-2, which are present in all the three different geographic populations. Although the distribution of the two mating types in three populations was not exactly equal, especially the Shanxi population had an obviously skewed distribution toward MAT1-2, but on the whole, the total ratio of the MAT1-1 and MAT1-2 was close to 1:1, indicating a positive association between the possibility of sexual reproduction among populations and genetic diversity (Dai et al., 2021). Such a premise seems to be contradictory with the moderate genetic diversity in three *S. turcica* populations as revealed in this study. It is possible that there are limitations in the use of SNP molecular markers for exploring the genetic diversity and population genetic structure of plant pathogens, which mandates cautious interpretations of the findings and warrants further investigation and refinement of the molecular tools used in such study.

There are relatively few studies about the pathogen of *S. turcica* that affects sorghum growth and development, this study represents the first attempt to use SNP molecular markers to analyze genetic diversity and population genetic structure of 87 *S. turcica* isolates from Henan, Shaanxi and Shanxi, the three provinces in China. Our results revealed relatively equal mating type distribution, moderate genetic diversity, and genetic differentiation among the three populations. Although careful interpretation is required for the results, there is no doubt that it will enrich our knowledge about the genetic diversity, population genetic structure of *S. turcica* isolates and provide theoretical bases for the disease management of sorghum NLB in the relevant regions.

## Conclusion

In this study, SNP molecular markers were developed to analyze the genetic diversity and population genetic structure of *S. turcica* for the first time. The *S. turcica* populations from three provinces in China are featured with moderate genetic diversity, high levels of gene flow among populations, and relatively equal mating type distribution of MAT1-1 and MAT1-2. The findings have improved our current understanding about the genetic diversity and population genetic structure of *S. turcica* in China.

## Data Availability Statement

The datasets presented in this study can be found in online repositories. The names of the repository/repositories and accession number(s) can be found at: NCBI BioProject–PRJNA798196.

## Author Contributions

YH designed the study. LC and JD performed all the experiments. JD and LZ analyzed the data and drafted the manuscript. TL reviewed and edited the manuscript. All authors contributed to the article and approved the submitted version.

## Funding

This work was supported by the grant U1804104 of the National Science Foundation of China, the grant CX(21)3022 of the Independent Innovation of Agricultural Sciences in Jiangsu Province and the grant BK20191236 of Jiangsu Science and Technology Development Program.

## Conflict of Interest

The authors declare that the research was conducted in the absence of any commercial or financial relationships that could be construed as a potential conflict of interest.

## Publisher’s Note

All claims expressed in this article are solely those of the authors and do not necessarily represent those of their affiliated organizations, or those of the publisher, the editors and the reviewers. Any product that may be evaluated in this article, or claim that may be made by its manufacturer, is not guaranteed or endorsed by the publisher.

## Footnotes

^1^ https://www.ncbi.nlm.nih.gov/sra/PRJNA803488

^2^ http://wheat.pw.usda.gov/demos/BatchPrimer3/

^3^ http://taylor0.biology.ucla.edu/structureHarvester/
