# Supplementary material for: Genetic Diversity and Population Genetic Structure of Setosphaeria turcica From Sorghum in Three Provinces of China Using Single Nucleotide Polymorphism Markers
Source: Front Microbiol. 2022 Mar 2;13:853202. doi: 10.3389/fmicb.2022.853202 (PMC8924674; doi:10.3389/fmicb.2022.853202)
Supplement: Supplementary file 1 [file Table_1.DOCX]

**Supplementary** **Table S1.** 77 SNP loci and primer pairs for *Setosphaeria turcica*

| Locus | Position | Forward Primer (5'-3') | Reverse Primer (5'-3') |
| --- | --- | --- | --- |
| L01*^1^ | 1-532966 | CACCGCACAGTCCTCAACTTT | TTAAAATTTGGCTTACCAGGACTTG |
| L02* | 1-1121603 | CTGTGAACACCGACGTCGTAAC | ATATGCCAAGCTGCAAGAACTGT |
| L03* | 1-1843365 | GTTTGGCCTGGTTGTCTATGTATTC | TACTAGGTATGTAGATGCGCGTGTG |
| L04* | 1-2799230 | TAGTTTAACGTCCTTTTTCAGCCC | GTAACAGGTAGCCATACACCTCCC |
| L05* | 2-150318 | CCGTACTTTACATGCAGACCACAT | ACAAATGTCCAAAATACGAACATCC |
| L06 | 2-1233702 | TAGCTGCGATCCACGAGTTAAGTA | GATGACTAGGCCATGTCGAAAAGT |
| L07* | 2-2081114 | AAAAGTAGTCGGGGGCTGTGTATAA | ATATATCGAATTGCATTATCGGGTG |
| L08* | 2-3138855 | CTAAGTTTAAAGAGGACGTGATTGT | TCAACGTTTAACAAAGGTACTAGGC |
| L09* | 3-44756 | ATCAGCTCCAGATATTGCAGAAAAA | TGATGTAGATCTGGGGTGATAGGAC |
| L10 | 3-976275 | TATATTAGCGTCTTCCATATTGCGG | AATTAACCAGGATGTCTCGGAGATT |
| L11* | 3-1847212 | ACATGGTTATCTGCTCAAAGGTCTC | CTCTGGCCAGTTTCCAAAGATATAC |
| L12 | 3-2767063 | GGTGTAATTTACTGCCCAGGATCT | GATAGCCTTTGTCGGCAGAATAGT |
| L13* | 4-395896 | AGAGAAAGAAGAAAGAAGAAAGAAGAGC | CTGTAGTTGTTAAACGTGGAAAAGACAT |
| L14* | 4-1247232 | TGCTCCTAATTGACCTAGCACTACC | TCTCTTGTAAACTAGTTCCGCAGGT |
| L15* | 4-1949754 | GACTGGGAGTGGATCAGTAACACTT | GAAAGTATGAATCTCTCTTGCCAGG |
| L16 | 4-2818621 | AGAGATAGAGTCACAAGGAACGCC | GACATCATTTCCTAGTGTGCGACT |
| L17 | 5-118908 | GTTAGGCTTAAGAGAGAAGGCACA | GACCTCCTAAACTTAAGAGCTTAGCC |
| L18* | 5-848589 | GCTGATACTAACACTACCACAGCCA | GAGATGCCCTTTTGCTTCTCC |
| L19* | 5-1420330 | CAACTATCAAAGCTCCAAGTTCCAT | TAGAGTTCTTACCAGCTGATGTCCC |
| L20 | 5-2306034 | TGAATCTCGCAAACTATTGCCTAC | TCTCCTAAGGCACTACAAGTTCTACC |
| L21 | 6-356581 | TGAATGTCAACGTCCAAACTCAAC | CTATAAGGGTGTGTGGCGGGTTAT |
| L22* | 6-983974 | GACAGCCTTACGGCAGCTACTATT | CTATCCTTGCTTGACGTCTCTTCTC |
| L23 | 6-1690306 | GCTCTTAGGTTTTCTTACGTAACTCTT | GCCATAGACAACGCTTAAAAGAT |
| L24 | 6-2272119 | GGCCTAAAAACCCTACACGTCTTAT | CAATATGTGTCTCCGTGTAGTTTCG |
| L25 | 7-418160 | CCTTCGTAGGCTTCTCTTAGTCCT | CTAGGGCACTGTTACCTAAGGGAC |
| L26* | 7-1359071 | GTCAGCTTAAGCCACGAACTCAT | CTTCAAATCCCTTAGAGAAGCCATC |
| L27 | 7-2101471 | ATCTGCCTAAGGACTGCGTTGTAT | TAGGCTAAAATGCTCGCCTAATTT |
| L28 | 8-311165 | CAAGTCTATGCCCCACTTATTCCT | AGACGTTGCGTATGCTATTTCCTA |
| L29* | 8-852741 | GATGGCAATGGGGCTTACTTAC | GTCACCGACTTTGACCTCACC |
| L30* | 8-1407070 | GGACGCAGAATAACAACATTAGGAG | TTTTGTTCTCTCGACCCATGTTT |
| L31 | 9-63972 | AATTCAACCTCGTGAGCTTTAACAC | AAGAGAGAAGCTTTCCGACATCATA |
| L32 | 9-549664 | AAAGTTTTGTCCCACGCTAATCTC | ACTTTAACGCCACAATTAAGGTGC |
| L33* | 9-1171207 | CCTTCTCTCCTCACACACAATCATA | GAGATAGCTATGTATGGTCACGCAG |
| L34 | 9-1664862 | GTTAAGAAGTGCTTGGGTAGGTCG | GCAGGCCTAAGAGAAAGAAGAATC |
| L35 | 10-504107 | TTCTACGTCTTCTGCTAGTTCAGC | GACTATTTAAAATCCTAGAGAAGGTAGG |
| L36* | 10-1052733 | CCGTACTTTACATGCAGACCACAT | ACAAATGTCCAAAATACGAACATCC |
| L37* | 10-1476856 | GCAACCTTTCTCCGACAAGTAAATA | CCGGAACGAGATTAGAGAAGTATCA |
| L38 | 10-1978944 | GCATGCATTTGATGACTAACGACT | ATGCTGTCGACCACACTCAACATA |
| L39* | 11-40790 | ATCAGCTCCAGATATTGCAGAAAAA | TGATGTAGATCTGGGGTGATAGGAC |
| L40* | 11-780809 | GCCTCTTAATTTCTCTAAGCGTAATAGC | GCAGCCCCTTACACTACGTAAGAA |
| L41 | 11-1660295 | TGGCTTTTTATGGTGTACAAGACC | CAGACCAAGTCAGTCTCGACGTAT |
| L42 | 12-459017 | CCCCCTACTTATGATCCAAGCTAA | CCCACATACGCCATGTAAAATAAA |
| L43 | 12-1366430 | CCTTAGTTTTGATGGTTTAAGAGGC | TAAGCTGTATTCGGTTATTTGCGA |
| L44 | 12-1629833 | CCTTGCTAAAGAAAGCTACTAGGCAC | AGAGGTTAAAAGCCTTACTGCTTGC |
| L45 | 13-198147 | CCTCTTATATACCTCGCGATTGCT | CGTACCTATTGCAGCACTAACTCTG |
| L46* | 13-878064 | CACAAATTGTCGGGCTATACCA | GTAAACATTTGTGATTCGAGCCAG |
| L47 | 13-1528663 | CTATTGGAGACGGTGATCGACAT | ATAAATCTGCGACACGGCTTTT |
| L48* | 14-310822 | AAGAATAACGCGATAAGCGAAGTC | GCTTGGCATCTCTACCTATTCGAT |
| L49 | 14-744997 | TACGGTAAGAAAAGATGTGCATGTG | AGACAAGTTTATTTAGCGTTTCCCC |
| L50* | 14-1206381 | AGCATGACGTTAAGCTGTGATAAAA | AGCAAAGAAACCTTCCTACTAGCG |
| L51 | 15-481165 | CAAAAGAAATTGTTGCGTCGTAAG | AAAAGACTAATCCAAAGTAGCCGC |
| L52 | 15-938529 | GGATAAGATTTGGTCTCGTACAACG | GCTCTGGGATTGGCTCAATACTT |
| L53 | 15-1342388 | TGCAGCATCACTATCTTGGAAACT | GTTTCATGTGCCATAACAACATCG |
| L54 | 16-248152 | GTTCTCTAAGCGTTTTGCTAGATG | GAGCTATGGTGGAAAAAGAGATCC |
| L55* | 16-748432 | GGATATTGGGGGTTTGAAGATTAAG | GATGAAAAACGTAGCGTATCCAAAC |
| L56 | 16-1240230 | AGGTGAATGCATACTCGTTAGTTAGG | TGCCTTATACAAAAGATCCTTACGC |
| L57 | 17-287484 | GGAGTAACCGGACTACCTTTTCAA | AGGCTTTCTGTTAGCTCGATCACT |
| L58* | 17-831816 | AAACGGCTACAAGATGGCG | ATAGCAAGTACAGCTGCAGAATGG |
| L59* | 17-1109559 | ATCATCATCATCATCATCAACACG | ACTAACGAACCGAAAGATTATGCAG |
| L60* | 18-111419 | TTTATTCCTCTCGTCCTCATCCTC | GTATCATTTCGGTTTTCCAGGTTC |
| L61* | 18-563251 | CTAGTATTGAAAACGAGTTACCCGC | GTACATGACGGGCTGTTTTTATTTT |
| L62* | 19-71784 | GGACAAGGTCACGCTAGTAAAGGT | TAGGTGAGATTATCGTTCGAGCAG |
| L63* | 19-407033 | GAGAAGGTTTCTCGCCCAGATAG | GCCCTAGTGCCTACCATCTACACTA |
| L64* | 19-779640 | ATTGAGGCTGTATCCCGCAGT | GCTGGTCCTCAGTCCAAAGTATCT |
| L65 | 20-131994 | ACAAACAAAACCAGCGTCACTTT | AACACCCCTCGTGTGTGATATTTT |
| L66* | 20-520160 | GTGAAGATGAAGAGCATGAGATCCT | AGTCTCCGCAGTTCTACAAGCAGAT |
| L67 | 20-829679 | AAAGCTGCGTACGCCTACTACTCT | GCCCCCTTCTTAAGCTAGTTAATTG |
| L68 | 21-14063 | GCGCGTTATTATCTATATATCAGCTCT | GACTAAAGCTAAGCTACTTTTATTGGAG |
| L69 | 21-458974 | TGCAGTCCAGTGATGTATGTATGC | GCCTGGAATAATGGGAGATTGTAG |
| L70* | 22-56000 | CGCTATTTAGAGCGTTCTTATGCC | TACCTTAGCAGGGGATGGATAGAC |
| L71 | 22-560832 | CGCTGCATGATCTAATTACAAAGG | GGGTTTTAGCTTCTCGATTGATCT |
| L72 | 23-87112 | GATGATCTTGCGACTTACTGCCTA | TGTTTTAGTTTGGCTCTCCCTCTT |
| L73 | 24-484964 | TTTTTGTACCAGTAGCAGCCGAC | CATTCAGACCTCGTAACCAAACAC |
| L74 | 26-263600 | TAAAGCACTTTGTCAAGCATACGG | GCTGGAGTAGGTGAGATGAGTGAG |
| L75 | 27-91353 | AGTCTTTTACTGTACGGTCCACCC | AAGAGAAGGTGATCAGAGCTTTCG |
| L76 | 28-48379 | TGGGCGTACTATTGGTTAATCCTT | ATAGCACTTCGCAAACAATCTCAC |
| L77 | 29-25741 | AAGCGCTTTGGTGTACATGCT | AGCTTACATTCACCGCACTCAAC |

^1^ These SNP loci with a asterisk were used to analyze genetic diversity of *S. turcica* in China
